# Supplementary material for: Atypical Sites of Lymphadenopathy after Anti-COVID-19 Vaccine: Ultrasound Features
Source: Medicina (Kaunas). 2022 Jan 27;58(2):197. doi: 10.3390/medicina58020197 (PMC8878753; doi:10.3390/medicina58020197)
Supplement: Supplementary file 1 [file medicina-58-00197-s001.zip › Table S1.pdf]

SEX	AGE	VACCINE	LYMPHO NODE LOCALIZATION	CLINICAL INSORGENCE	ONCOLOGICAL HISTORY	NM	SIZE	FORM	SONOELASTO	SMI	US-FOLLOW-UP TO 2 WEEKS
W	25	PFIZER	AXILLARY IPSILATERAL TO VACCINE INJECTION	AFTER 3 DAY TO FIRST DOSE VACCINE REFERRED AXILLARY SWELLING AND PAIN. ALSO HYPOMOBILITY IPSILATERAL ARM	NONE	6	VARIABLE: FROM 0,7 CM TO 2,8 CM.	OVALAR	PREVALENT HARD PATTERN	CENTRAL AND PERIPHERAL VASCULAR SIGNALS	NORMALI ZED TO 60 DAYS
M	64	PFIZER	SUPRACLAVICULAR IPSILATERAL TO VACCINE INJECTION	OCCASIONALLY AUTOPALPATION 2 WEEKS AFTER 2 DOSE VACCINE	NONE	2	ABOUT 1 CM	OVALAR	STIFFNESS SIMILAR TO SURROUNDING TISSUE	NORMAL	UNNECESS ARY OTHER FOLLOW-UP
W	28	ASTAZENECA	SUPRACLAVICULAR IPSILATERAL TO VACCINE INJECTION	AFTER 2 DAY TO FIRST DOSE VACCINE REFERRED SUPRACLAVICULAR SWELLING AND PAIN	NONE	3	VARIABLE: from 0,6 to 1,5 cm..	OVALAR	PREVALENT HARD PATTERN	CENTRAL AND PERIPHERAL VASCULAR SIGNALS	NORMALI ZED TO 45 DAYS
W	72	ASTAZENECA	SUPRACLAVICULAR IPSILATERAL TO VACCINE INJECTION	AFTER 1 DAY TO FIRST DOSE VACCINE REFERRED SUPRACLAVICULAR SWELLING AND PAIN.	NONE	2	SUBCENTIME TRIC SIZE	ROUND	PREVALENT HARD PATTERN	PERIPHERAL VASCULAR SIGNALS	NORMALI ZED TO 30 DAYS
M	42	PFIZER	AXILLARY IPSILATERAL TO VACCINE INJECTION	OCCASIONALLY 1 WEEKS AFTER SECOND DOSE DURING CHEST CT	NONE	3	VARIABLE: FROM 1,5 TO 2 CM.	OVALAR	STIFFNESS SIMILAR TO SURROUNDING TISSUE	NORMAL	UNNECESS ARY OTHER FOLLOW-UP
M	39	ASTAZENECA	SUPRACLAVICULAR IPSILATERAL TO VACCINE INJECTION	AFTER 4 DAY TO FIRST DOSE VACCINE REFERRED SUPRACLAVICULAR SWELLING AND PAIN	NONE	1	SUBCENTIME TRIC SIZE	ROUND	PREVALENT HARD PATTERN	CENTRAL AND PERIPHERAL VASCULAR SIGNALS	NORMALI ZED TO 30 DAYS
M	60	PFIZER	SUPRACLAVICULAR IPSILATERAL TO VACCINE INJECTION	OCCASIONALLY AUTOPALPATION 12 DAYS AFTER 2 DOSE VACCINE	NONE	2	ABOUT 1 CM	OVALAR	STIFFNESS SIMILAR TO SURROUNDING TISSUE	CENTRAL AND PERIPHERAL VASCULAR SIGNALS	UNNECESS ARY OTHER FOLLOW-UP
W	49	PFIZER	AXILLARY IPSILATERAL TO VACCINE INJECTION	OCCASIONALLY, AFTER 6 DAYS FROM FIRST DOSE, DURING BREAST SONOGRAPHY FOR ONCOLOGICAL SURVEILLANCE	BREAST CANCER 3 YEARS AGO	4	VARIABLE: FROM 1 TO 2 CM.	OVALAR	STIFFNESS SIMILAR TO SURROUNDING TISSUE	CENTRAL AND PERIPHERAL VASCULAR SIGNALS	UNNECESS ARY OTHER FOLLOW-UP

M	41	MODERNA	SUPRACLAVICULAR IPSILATERAL TO VACCINE INJECTION	AFTER 2 DAY TO FIRST DOSE VACCINE REFERRED SUPRACLAVICULAR SWELLING AND PAIN.	NONE	3	SUBCENTIMETRIC SIZE	OVALAR	STIFFNESS SIMILAR TO SURROUNDING TISSUE	CENTRAL AND PERIPHERAL VASCULAR SIGNALS	NORMALIZED TO 30 DAYS
W	54	PFIZER	AXILLARY IPSILATERAL TO VACCINE INJECTION	OCCASIONALLY, AFTER 14 DAYS FROM SECOND DOSE, DURING BREAST SONOGRAPHY FOR SURVEILLANCE	NONE	3	VARIABLE: FROM 1 TO 2 CM.	OVALAR	STIFFNESS SIMILAR TO SURROUNDING TISSUE	NORMAL	UNNECESSARY OTHER FOLLOW-UP
W	74	ASTAZENECA	SUPRACLAVICULAR IPSILATERAL TO VACCINE INJECTION	AFTER 10 DAY TO FIRST DOSE VACCINE.	NONE	2	SUBCENTIMETRIC SIZE	OVALAR	STIFFNESS SIMILAR TO SURROUNDING TISSUE	NORMAL	UNNECESSARY OTHER FOLLOW-UP
M	35	PFIZER	SUPRACLAVICULAR IPSILATERAL TO VACCINE INJECTION	DAY AFTER THE FIRST DOSE VACCINE REFERRED SUPRACLAVICULAR SWELLING AND PAIN	NONE	1	ABOUT 1,5 CM	ROUND	PREVALENT HARD PATTERN	CENTRAL AND PERIPHERAL VASCULAR SIGNALS	NORMALIZED TO 60 DAYS
W	52	PFIZER	AXILLARY IPSILATERAL TO VACCINE INJECTION	OCCASIONALLY DURING BREAST SONOGRAPHY FOR ONCOLOGICAL SURVEILLANCE	NONE	3	VARIABLE: FROM 1 TO 2,5 CM.	OVALAR	STIFFNESS SIMILAR TO SURROUNDING TISSUE	NORMAL	UNNECESSARY OTHER FOLLOW-UP
W	26	ASTAZENECA	SUPRACLAVICULAR IPSILATERAL	2 DAYS AFTER THE FIRST DOSE VACCINE REFERRED AXILLARY SWELLING AND PAIN	NONE	5	SUBCENTIMETRIC SIZE	OVALAR	PREVALENT HARD PATTERN	CENTRAL AND PERIPHERAL VASCULAR SIGNALS	NORMALIZED TO 45 DAYS
W	53	PFIZER	AXILLARY IPSILATERAL TO VACCINE INJECTION	OCCASIONALLY, AFTER 16 DAYS FROM FIRST DOSE, DURING BREAST SONOGRAPHY FOR ONCOLOGICAL SURVEILLANCE	BREAST CANCER 2 YEARS AGO	3	VARIABLE: FROM 1 TO 2 CM.	OVALAR	STIFFNESS SIMILAR TO SURROUNDING TISSUE	NORMAL	UNNECESSARY OTHER FOLLOW-UP
M	62	PFIZER	AXILLARY IPSILATERAL TO VACCINE INJECTION	OCCASIONALLY 2 WEEKS AFTER FIRST DOSE DURING CHEST CT TO MONITORING SMALL POLMONARY NODULES	NONE	3	VARIABLE: FROM 1,5 TO 2 CM.	OVALAR	STIFFNESS SIMILAR TO SURROUNDING TISSUE	NORMAL	UNNECESSARY OTHER FOLLOW-UP
M	57	PFIZER	AXILLARY IPSILATERAL TO VACCINE INJECTION	OCCASIONALLY AUTOPALPATION 2 WEEKS AFTER 2 DOSE VACCINE	KIDNEY CANCER 4 YEARS AGO	2	ABOUT 1 CM	OVALAR	STIFFNESS SIMILAR TO SURROUNDING TISSUE	NORMAL	NORMALIZED TO 30 DAYS
W	69	ASTAZENECA	SUPRACLAVICULAR IPSILATERAL TO VACCINE INJECTION	AFTER 3 DAY TO FIRST DOSE VACCINE.	NONE	3	SUBCENTIMETRIC SIZE	ROUND	STIFFNESS SIMILAR TO SURROUNDING TISSUE	CENTRAL AND PERIPHERAL	UNNECESSARY OTHER

										VASCULAR SIGNALS	FOLLOW-UP
W	37	PFIZER	AXILLARY IPSILATERAL TO VACCINE INJECTION	AFTER 3 DAY TO FIRST DOSE VACCINE REFERRED AXILLARY SWELLING	MELANOMA 5 YEARS AGO	5	VARIABLE: FROM 1,5 TO 2 CM.	OVALAR	PREVALENT HARD PATTERN	CENTRAL AND PERIPHERAL VASCULAR SIGNALS	NORMALIZED TO 45 DAYS
M	63	MODERNA	AXILLARY IPSILATERAL TO VACCINE INJECTION	OCCASIONALLY 16 DAYS AFTER FIRST DOSE DURING MAMMOGRAPHY	NONE	3	VARIABLE: FROM 1,5 TO 2 CM.	OVALAR	STIFFNESS SIMILAR TO SURROUNDING TISSUE	NORMAL	UNNECESSARY OTHER FOLLOW-UP
F	32	ASTRAZENECA	SUPRACLAVICULAR IPSILATERAL TO VACCINE INJECTION	AFTER DAY TO FIRST DOSE VACCINE REFERRED SUPRACLAVICULAR SWELLING AND PAIN	NONE	1	ABOUT 1,2 CM	ROUND	PREVALENT HARD PATTERN	CENTRAL AND PERIPHERAL VASCULAR SIGNALS	NORMALIZED TO 30 DAYS
F	29	PFIZER	SUPRACLAVICULAR IPSILATERAL TO VACCINE INJECTION	AFTER DAY TO FIRST DOSE VACCINE REFERRED SUPRACLAVICULAR SWELLING AND PAIN	NONE	2	SUBCENTIMETRIC	ROUND	PREVALENT HARD PATTERN	CENTRAL AND PERIPHERAL VASCULAR SIGNALS	NORMALIZED TO 30 DAYS
F	66	MODERNA	SUPRACLAVICULAR IPSILATERAL TO VACCINE INJECTION	OCCASIONALLY 2 WEEKS AFTER SECOND DOSE DURING SHOULDER RM	NONE	2	ABOUT 1 CM	OVALAR	STIFFNESS SIMILAR TO SURROUNDING TISSUE	CENTRAL AND PERIPHERAL VASCULAR SIGNALS	NORMALIZED TO 30 DAYS
M	59	ASTRAZENECA	SUPRACLAVICULAR IPSILATERAL TO VACCINE INJECTION	OCCASIONALLY AUTOPALPATION 4 DAYS AFTER 2 DOSE VACCINE	NONE	2	0,7 AND 1,2 CM	OVALAR	STIFFNESS SIMILAR TO SURROUNDING TISSUE	NORMAL	UNNECESSARY OTHER FOLLOW-UP
W	37	PFIZER	AXILLARY IPSILATERAL TO VACCINE INJECTION	AFTER 3 DAY TO FIRST DOSE VACCINE REFERRED AXILLARY SWELLING	MELANOMA 5 YEARS AGO	5	VARIABLE: FROM 1,5 TO 2 CM.	OVALAR	PREVALENT HARD PATTERN	CENTRAL AND PERIPHERAL VASCULAR SIGNALS	NORMALIZED TO 45 DAYS
F	58	MODERNA	AXILLARY IPSILATERAL TO VACCINE INJECTION	OCCASIONALLY 10 DAYS AFTER FIRST DOSE DURING MAMMOGRAPHY	NONE	4	VARIABLE: FROM 1 TO 2,5 CM.	OVALAR	STIFFNESS SIMILAR TO SURROUNDING TISSUE	NORMAL	UNNECESSARY OTHER FOLLOW-UP
F	73	ASTRAZENECA	SUPRACLAVICULAR IPSILATERAL TO VACCINE INJECTION	AFTER DAY TO FIRST DOSE VACCINE REFERRED SUPRACLAVICULAR SWELLING	NONE	2	ABOUT 1 CM	ROUND	PREVALENT HARD PATTERN	CENTRAL AND PERIPHERAL VASCULAR SIGNALS	NORMALIZED TO 75 DAYS
F	31	MODERNA	SUPRACLAVICULAR IPSILATERAL TO VACCINE INJECTION	AFTER 3 DAYs TO FIRST DOSE VACCINE REFERRED SUPRACLAVICULAR SWELLING AND PAIN	NONE	3	ABOUT 1 CM	OVALAR	STIFFNESS SIMILAR TO SURROUNDING TISSUE	CENTRAL AND PERIPHERAL VASCULAR SIGNALS	NORMALIZED TO 30 DAYS

F	65	PFIZER	SUPRACLAVICULAR IPSILATERAL TO VACCINE INJECTION	AFTER 5 DAYS TO FIRST DOSE VACCINE REFERRED SUPRACLAVICULAR SWELLING	NONE	2	ABOUT 1 CM	OVALAR	STIFFNESS SIMILAR TO SURROUNDING TISSUE	CENTRAL AND PERIPHERAL VASCULAR SIGNALS	NORMALIZED TO 30 DAYS
M	61	ASTRAZENECA	SUPRACLAVICULAR IPSILATERAL	OCCASIONALLY AUTOPALPATION 3 DAYS AFTER 2 DOSE VACCINE	NONE	2	1 AND 1,5 CM	OVALAR	STIFFNESS SIMILAR TO SURROUNDING TISSUE	CENTRAL AND PERIPHERAL VASCULAR SIGNALS	NORMALIZED TO 30 DAYS
W	36	PFIZER	AXILLARY IPSILATERAL TO VACCINE INJECTION	AFTER 3 DAY TO FIRST DOSE VACCINE REFERRED AXILLARY SWELLING	NONE	4	VARIABLE: FROM 1 TO 2 CM.	OVALAR	STIFFNESS SIMILAR TO SURROUNDING TISSUE	CENTRAL AND PERIPHERAL VASCULAR SIGNALS	NORMALIZED TO 45 DAYS
M	58	MODERNA	AXILLARY IPSILATERAL TO VACCINE INJECTION	OCCASIONALLY 13 DAYS AFTER FIRST DOSE DURING MAMMOGRAPHY	NONE	3	VARIABLE: FROM 1,5 TO 2,5 CM.	OVALAR	STIFFNESS SIMILAR TO SURROUNDING TISSUE	NORMAL	NORMALIZED TO 45 DAYS
F	62	ASTRAZENECA	SUPRACLAVICULAR IPSILATERAL TO VACCINE INJECTION	AFTER DAY TO FIRST DOSE VACCINE REFERRED SUPRACLAVICULAR SWELLING AND PAIN	NONE	1	ABOUT 1,5 CM	ROUND	PREVALENT HARD PATTERN	CENTRAL AND PERIPHERAL VASCULAR SIGNALS	NORMALIZED TO 30 DAYS
M	27	PFIZER	SUPRACLAVICULAR IPSILATERAL TO VACCINE INJECTION	AFTER DAY TO FIRST DOSE VACCINE REFERRED SUPRACLAVICULAR SWELLING AND PAIN	NONE	2	SUBCENTIMETRIC	ROUND	PREVALENT HARD PATTERN	CENTRAL AND PERIPHERAL VASCULAR SIGNALS	NORMALIZED TO 30 DAYS
M	48	MODERNA	SUPRACLAVICULAR IPSILATERAL TO VACCINE INJECTION	AFTER 2 DAYS AFTER SECOND DOSE VACCINE REFERRED SUPRACLAVICULAR SWELLING	NONE	2	ABOUT 1 CM	OVALAR	STIFFNESS SIMILAR TO SURROUNDING TISSUE	CENTRAL AND PERIPHERAL VASCULAR SIGNALS	NORMALIZED TO 30 DAYS
F	45	ASTRAZENECA	SUPRACLAVICULAR IPSILATERAL TO VACCINE INJECTION	OCCASIONALLY AUTOPALPATION 3 DAYS AFTER 2 DOSE VACCINE	NONE	2	1 AND 1,5 CM	OVALAR	STIFFNESS SIMILAR TO SURROUNDING TISSUE	NORMAL	NORMALIZED TO 30 DAYS
F	38	PFIZER	AXILLARY IPSILATERAL TO VACCINE INJECTION	AFTER 3 DAY TO FIRST DOSE VACCINE REFERRED AXILLARY SWELLING	NONE	5	VARIABLE: FROM 1,5 TO 2 CM.	OVALAR	STIFFNESS SIMILAR TO SURROUNDING TISSUE	CENTRAL AND PERIPHERAL VASCULAR SIGNALS	NORMALIZED TO 30 DAYS
M	53	MODERNA	AXILLARY IPSILATERAL TO VACCINE INJECTION	OCCASIONALLY 10 DAYS AFTER FIRST DOSE DURING MAMMOGRAPHY	NONE	3	VARIABLE: FROM 1 TO 2 CM.	OVALAR	STIFFNESS SIMILAR TO SURROUNDING TISSUE	NORMAL	UNNECESSARY OTHER FOLLOW-UP
M	42	ASTRAZENECA	SUPRACLAVICULAR IPSILATERAL TO VACCINE INJECTION	AFTER 2 DAYS TO SECOND DOSE VACCINE REFERRED SUPRACLAVICULAR SWELLING AND PAIN	NONE	1	ABOUT 1 CM	ROUND	PREVALENT HARD PATTERN	CENTRAL AND PERIPHERAL VASCULAR SIGNALS	NORMALIZED TO 45 DAYS

M	27	PFIZER	SUPRACLAVICULAR IPSILATERAL TO VACCINE INJECTION	AFTER DAY TO FIRST DOSE VACCINE REFERRED SUPRACLAVICULAR SWELLING AND PAIN	NONE	3	SUBCENTIMETRIC	ROUND	PREVALENT HARD PATTERN	CENTRAL AND PERIPHERAL VASCULAR SIGNALS	NORMALIZED TO 45 DAYS
F	46	MODERNA	SUPRACLAVICULAR IPSILATERAL TO VACCINE INJECTION	AFTER DAY TO SECOND DOSE VACCINE REFERRED SUPRACLAVICULAR SWELLING AND PAIN	NONE	2	ABOUT 1 CM	OVALAR	STIFFNESS SIMILAR TO SURROUNDING TISSUE	CENTRAL AND PERIPHERAL VASCULAR SIGNALS	NORMALIZED TO 30 DAYS
M	49	ASTRAZENECA	SUPRACLAVICULAR IPSILATERAL TO VACCINE INJECTION	OCCASIONALLY AUTOPALPATION 3 DAYS AFTER 2 DOSE VACCINE	NONE	2	ABOUT 1 CM	OVALAR	STIFFNESS SIMILAR TO SURROUNDING TISSUE	CENTRAL AND PERIPHERAL VASCULAR SIGNALS	NORMALIZED TO 30 DAYS
M	39	PFIZER	AXILLARY IPSILATERAL TO VACCINE INJECTION	AFTER 3 DAYS TO FIRST DOSE VACCINE REFERRED AXILLARY SWELLING	NONE	5	VARIABLE: FROM 1 TO 2 CM.	OVALAR	STIFFNESS SIMILAR TO SURROUNDING TISSUE	CENTRAL AND PERIPHERAL VASCULAR SIGNALS	NORMALIZED TO 45 DAYS
F	53	MODERNA	AXILLARY IPSILATERAL TO VACCINE INJECTION	OCCASIONALLY 12 DAYS AFTER FIRST DOSE DURING MAMMOGRAPHY	NONE	2	1,5 AND 2 CM.	OVALAR	STIFFNESS SIMILAR TO SURROUNDING TISSUE	NORMAL	UNNECESSARY OTHER FOLLOW-UP
M	33	PFIZER	SUPRACLAVICULAR IPSILATERAL TO VACCINE INJECTION	AFTER DAY TO FIRST DOSE VACCINE REFERRED SUPRACLAVICULAR SWELLING AND PAIN	NONE	1	ABOUT 1 CM	ROUND	PREVALENT HARD PATTERN	CENTRAL AND PERIPHERAL VASCULAR SIGNALS	NORMALIZED TO 30 DAYS
M	30	PFIZER	SUPRACLAVICULAR IPSILATERAL	AFTER 5 DAYS TO FIRST DOSE VACCINE REFERRED SUPRACLAVICULAR SWELLING	NONE	2	SUBCENTIMETRIC	OVALAR	STIFFNESS SIMILAR TO SURROUNDING TISSUE	CENTRAL AND PERIPHERAL VASCULAR SIGNALS	UNNECESSARY OTHER FOLLOW-UP
M	37	PFIZER	AXILLARY IPSILATERAL TO VACCINE INJECTION	AFTER 3 DAY TO FIRST DOSE VACCINE REFERRED AXILLARY SWELLING	NONE	5	VARIABLE: FROM 1 TO 2,5 CM.	OVALAR	STIFFNESS SIMILAR TO SURROUNDING TISSUE	CENTRAL AND PERIPHERAL VASCULAR SIGNALS	NORMALIZED TO 45 DAYS
W	65	PFIZER	AXILLARY IPSILATERAL TO VACCINE INJECTION	OCCASIONALLY 10 DAYS AFTER SECOND DOSE DURING CHEST CT	NONE	5	VARIABLE: FROM 1,5 TO 2 CM.	OVALAR	STIFFNESS SIMILAR TO SURROUNDING TISSUE	NORMAL	NORMALIZED TO 30 DAYS
W	28	PFIZER	AXILLARY IPSILATERAL TO VACCINE INJECTION	AFTER 3 DAY TO FIRST DOSE VACCINE REFERRED AXILLARY SWELLING AND PAIN. ALSO HYPOMOBILITY IPSILATERAL ARM	NONE	6	VARIABLE: FROM 0,7 CM TO 2,8 CM.	OVALAR	PREVALENT HARD PATTERN	CENTRAL AND PERIPHERAL VASCULAR SIGNALS	NORMALIZED TO 60 DAYS
W	44	MODERNA	SUPRACLAVICULAR IPSILATERAL TO VACCINE INJECTION	OCCASIONALLY AUTOPALPATION 2	NONE	2	SUBCENTIMETRIC SIZE	OVALAR	STIFFNESS SIMILAR TO SURROUNDING TISSUE	NORMAL	UNNECESSARY OTHER

				WEEKS AFTER 2 DOSE VACCINE							FOLLOW-UP
W	33	ASTAZENECA	SUPRACLAVICULAR IPSILATERAL TO VACCINE INJECTION	AFTER 3 DAYS TO SECOND DOSE VACCINE REFERRED AXILLARY SWELLING	NONE	4	VARIABLE: FROM 1 TO 1,5 cm..	OVALAR	STIFFNESS SIMILAR TO SURROUNDING TISSUE	CENTRAL AND PERIPHERAL VASCULAR SIGNALS	NORMALIZED TO 45 DAYS
W	68	ASTAZENECA	SUPRACLAVICULAR IPSILATERAL	AFTER 1 DAY TO FIRST DOSE VACCINE REFERRED SUPRACLAVICULAR SWELLING AND PAIN.	NONE	2	SUBCENTIMETRIC SIZE	ROUND	PREVALENT HARD PATTERN	PERIPHERAL VASCULAR SIGNALS	NORMALIZED TO 45 DAYS
M	45	MODERNA	AXILLARY IPSILATERAL TO VACCINE INJECTION	OCCASIONALLY 2 WEEKS AFTER SECOND DOSE DURING CHEST CT	NONE	3	VARIABLE: FROM 1,5 TO 2 CM.	OVALAR	STIFFNESS SIMILAR TO SURROUNDING TISSUE	NORMAL	UNNECESSARY OTHER FOLLOW-UP
M	40	ASTAZENECA	SUPRACLAVICULAR IPSILATERAL TO VACCINE INJECTION	AFTER 4 DAY TO SECOND DOSE VACCINE REFERRED SUPRACLAVICULAR SWELLING AND PAIN	NONE	1	ABOUT 1 CM	ROUND	PREVALENT HARD PATTERN	CENTRAL AND PERIPHERAL VASCULAR SIGNALS	NORMALIZED TO 45 DAYS
W	23	PFIZER	AXILLARY IPSILATERAL TO VACCINE INJECTION	AFTER 2 DAYS TO FIRST DOSE VACCINE REFERRED AXILLARY SWELLING AND PAIN. ALSO HYPOMOBILITY IPSILATERAL ARM	NONE	3	VARIABLE: FROM 1 CM TO 2,5 CM.	OVALAR	STIFFNESS SIMILAR TO SURROUNDING TISSUE	CENTRAL AND PERIPHERAL VASCULAR SIGNALS	NORMALIZED TO 60 DAYS
M	44	PFIZER	SUPRACLAVICULAR IPSILATERAL TO VACCINE INJECTION	OCCASIONALLY AUTOPALPATION 2 WEEKS AFTER 2 DOSE VACCINE	NONE	2	ABOUT 1 CM	OVALAR	STIFFNESS SIMILAR TO SURROUNDING TISSUE	NORMAL	UNNECESSARY OTHER FOLLOW-UP
W	38	MODERNA	SUPRACLAVICULAR IPSILATERAL TO VACCINE INJECTION	AFTER 2 DAYS TO FIRST DOSE VACCINE REFERRED SUPRACLAVICULAR SWELLING AND PAIN	NONE	2	1 AND 1,5 CM.	OVALAR	PREVALENT HARD PATTERN	CENTRAL AND PERIPHERAL VASCULAR SIGNALS	NORMALIZED TO 30 DAYS
W	70	ASTAZENECA	SUPRACLAVICULAR IPSILATERAL TO VACCINE INJECTION	AFTER 1 DAY TO FIRST DOSE VACCINE REFERRED SUPRACLAVICULAR SWELLING AND PAIN.	NONE	2	ABOUT 1 CM	ROUND	PREVALENT HARD PATTERN	PERIPHERAL VASCULAR SIGNALS	NORMALIZED TO 60 DAYS
M	47	PFIZER	AXILLARY IPSILATERAL TO VACCINE INJECTION	OCCASIONALLY 2 WEEKS AFTER SECOND DOSE DURING CHEST CT	NONE	5	VARIABLE: FROM 1,5 TO 2 CM.	OVALAR	STIFFNESS SIMILAR TO SURROUNDING TISSUE	NORMAL	NORMALIZED TO 30 DAYS
W	20	MODERNA	SUBCLAVICULAR IPSILATERAL TO VACCINE INJECTION	AFTER 3 DAYS TO FIRST DOSE VACCINE REFERRED	NONE	2	0,5 AND 1 CM.	OVALAR	STIFFNESS SIMILAR TO SURROUNDING TISSUE	CENTRAL AND PERIPHERAL VASCULAR SIGNALS	NORMALIZED TO 30 DAYS

				SUBCLAVICULAR SWELLING							
M	21	MODERNA	ARM IPSILATERAL TO VACCINE INJECTION	AFTER 2 DAYS TO FIRST DOSE VACCINE REFERRED ARM SWELLING	NONE	1	ABOUT 1 CM	OVALAR	STIFFNESS SIMILAR TO SURROUNDING TISSUE	CENTRAL AND PERIPHERAL VASCULAR SIGNALS	NORMALI ZED TO 45 DAYS
W	23	PFIZER	PECTORIAL MUSCLE	AFTER 3 DAYS TO FIRST DOSE VACCINE REFERRED PECTORIAL SWELLING	NONE	1	ABOUT 1 CM	OVALAR	STIFFNESS SIMILAR TO SURROUNDING TISSUE	NORMAL	UNNECESS ARY OTHER FOLLOW-UP
M	37	MODERNA	IPSILATERAL TO VACCINE INJECTION	AFTER 3 DAYS TO FIRST DOSE VACCINE REFERRED NUCAL SWELLING	NONE	1	ABOUT 1 CM	OVALAR	PREVALENT HARD PATTERN	CENTRAL AND PERIPHERAL VASCULAR SIGNALS	NORMALI ZED TO 30 DAYS
W	63	ATRAZENACA	SUBCLAVICULAR IPSILATERAL TO VACCINE INJECTION	AFTER 4 DAYS TO FIRST DOSE VACCINE REFERRED SUBCLAVICULAR SWELLING	NONE	1	ABOUT 1 CM	OVALAR	STIFFNESS SIMILAR TO SURROUNDING TISSUE	CENTRAL AND PERIPHERAL VASCULAR SIGNALS	NORMALI ZED TO 30 DAYS
